# Hybrid institutional complexes in global governance

**DOI:** 10.1007/s11558-021-09431-3

**Published:** 2021-05-26

**Authors:** Kenneth W. Abbott, Benjamin Faude

**Affiliations:** 1 Arizona State University, Tempe, AZ USA; 2 Department of Government, Department of International Relations, London School of Economics and Political Science (LSE), England, WC2A 2AE UK

**Keywords:** Regime complex, Informal institutions, Transnational institutions, Institutional diversity, Functional differentiation, Informal hierarchy, Institutional fit, Y8 Related Disciplines

## Abstract

Most issue areas in world politics today are governed neither by individual institutions nor by regime complexes composed of formal interstate institutions. Rather, they are governed by “hybrid institutional complexes” (HICs) comprising heterogeneous interstate, infra-state, public–private and private transnational institutions, formal and informal. We develop the concept of the HIC as a novel descriptive and analytical lens for the study of contemporary global governance. The core structural difference between HICs and regime complexes is the greater diversity of institutional forms within HICs. Because of that diversity, HICs operate differently than regime complexes in two significant ways: (1) HICs exhibit relatively greater functional differentiation among their component institutions, and hence suffer from relatively fewer overlapping claims to authority; and (2) HICs exhibit greater informal hierarchy among their component institutions, and hence benefit from greater ordering. Both are systemic features. HICs have characteristic governance benefits: they offer good “substantive fit” for multi-faceted governance problems and good “political fit” for the preferences of diverse constituents; constrain conflictive cross-institutional strategies; and are conducive to mechanisms of coordination, which enhance substantive coherence. Yet HICs also pose characteristic governance risks: individual institutions may take on aspects of problems for which they are ill-suited; multiple institutions may create confusion; HICs can amplify conflict and contestation rather than constraining them; and the “soft” institutions within HICs can reduce the focality of incumbent treaties and intergovernmental organizations and forestall the establishment of new ones. We outline a continuing research agenda for exploring the structures, operations and governance implications of HICs.

## Introduction

Most issue areas in world politics today are governed neither by individual institutions nor by regime complexes composed of formal interstate institutions. Rather, they are governed by “hybrid institutional complexes” (HICs) comprising heterogeneous interstate, infra-state, public–private and private transnational institutions, formal and informal. HICs are widespread in contemporary global governance; they can be observed in numerous issue areas including, for example, climate change; global health; financial regulation; cyberspace; and nuclear safety, all discussed further below.^1^

HICs include not only the multilateral treaties and formal intergovernmental organizations (FIGOs) that the literature treats as comprising regime complexes. They also include additional institutional forms in varied combinations: informal inter-governmental organizations (IIGOs) (Vabulas & Snidal, 2013, 2020); trans-governmental networks (TGNs) (Slaughter, 2004); transnational public–private partnerships (TPPPs) (Andonova, 2010, 2017; Reinsberg & Westerwinter, 2021); transnational associations of sub-national governments (Betsill & Bulkeley, 2006; Gordon, 2020); private transnational regulatory organizations (PTROs) (Abbott et al., 2016); and other types of institutions.

HICs are not, in most cases, free-standing complexes that operate separately from interstate regime complexes within the same domains.^2^ Rather, HICs have emerged as IIGOs, PTROs and other alternative institutional forms have proliferated during the past three decades (Pauwelyn et al., 2012), most often in domains already occupied by regime complexes.^3^ This process effectively transmuted incumbent regime complexes into HICs, modifying the international governance architecture.^4^

During those decades, for example, the number of IIGOs rose from 27 to 72 (Vabulas & Snidal, 2013) or even more (Vabulas & Snidal, 2020; Westerwinter et al., 2021), at least a 167% increase; IIGOs now “constitute as much as a third of all the currently active international organizations” (Roger, 2020: 8). TPPPs increased from 63 to 435, nearly a 600% increase (Westerwinter et al., 2021), while TGNs expanded from 25 to 141, a 464% increase (Abbott et al., 2018: 2).^5^ This explosion of institutional diversity – in issue areas where interstate regime complexes were already prevalent – has made HICs the most common type of global governance complex (Eilstrup-Sangiovanni & Westerwinter, 2020).

IR scholarship has responded to these developments with an increased focus on institutional *density*, moving from the study of individual institutions to analyses of institutional interactions, regime complexes, and cross-institutional strategies (Alter & Meunier, 2009; Gehring & Oberthür, 2009; Jupille et al., 2013; Young, 2002). However, it has lagged the growth in institutional *diversity*, continuing to focus primarily on formal, legalized interstate institutions (e.g., Alter & Raustiala, 2018; Gehring & Faude, 2014; Keohane & Victor, 2011), excluding other institutional forms from its purview.

Robust parallel literatures on transnational and informal institutions have simultaneously developed. The transnational literature emphasizes the governance roles played by private and sub-state actors and institutions (e.g., Pauwelyn et al., 2012; Abbott, 2012; Bulkeley et al., 2014), sometimes including their interactions with interstate institutions (e.g., Green, 2014; Green & Auld, 2017; Karassin & Perez, 2020); the informality literature documents the growing importance of less formalized and legalized arrangements such as IIGOs and TGNs (Roger, 2020; Westerwinter et al., 2021).

However, IR scholarship has yet to develop an analytical lens that encompasses all of these institutional forms (cf. Kahler, 2016; Lake, 2020; Ruggie, 2014). That is, it has yet to systematically analyze the distinctive structures, operations, governance benefits and risks of governance complexes populated by diverse institutional forms.^6^ Because the literatures on regime complexes, transnational and informal institutions each exclude major categories of institutional forms, none of them alone can produce a complete understanding of contemporary global governance.

To facilitate more integrated analysis, we introduce the concept of the hybrid institutional complex (HIC) as a novel descriptive and analytical lens. In Sect. 2, we describe prominent HICs and develop the HIC concept in relation to the well-established concept of the regime complex. Both HICs and regime complexes are global governance complexes composed of governance institutions. The core structural difference between them is the greater diversity of institutional forms within HICs.

Because of that diversity, we posit, HICs operate differently than regime complexes in two significant ways: (1) HICs exhibit relatively greater functional differentiation among their component institutions, and hence suffer from relatively fewer overlapping claims to authority; and (2) HICs exhibit greater informal hierarchy among their component institutions, and hence benefit from greater ordering. Both are systemic features, not features of individual institutions; both reduce institutional duplication, norm collision and instability, outcomes central to the regime complex literature.

In Sect. 3, we address the continuing possibility of discord and conflict within HICs. Epistemic and normative differences, cross-institutional strategies, and strategies of contestation can all exist within HICs, driven by the interests and values of the institutions themselves or of their members – e.g., (powerful) states, agencies, or business firms. Yet the specific structural features of HICs – institutional diversity, functional differentiation and informal hierarchy – constrain these strategies to some extent and dampen their harmful effects.

In Sect. 4, we analyze major implications of HICs for governance outcomes, highlighting both positive and negative effects. On one hand, the structural features of HICs produce characteristic systemic *benefits* for governance. First, HICs offer good “substantive fit” for multi-faceted governance problems. In principle, functional differentiation allows each institutional form to address those aspects of a problem to which it is best suited, and to reinforce one another by addressing common problems in complementary ways. Second, HICs offer good “political fit” for the preferences of diverse constituents and stakeholders. Within limits, actors can choose to participate in those institutions whose benefits, weaknesses, costs and risks most closely match their characteristics and preferences, increasing the incentive-compatibility of cooperation. Third, although coordination mechanisms are driven by individual actors and institutions, not governance systems, the functional differentiation and informal hierarchy of HICs make them *conducive* to coordination, producing relatively strong substantive coherence.

On the other hand, the structural features of HICs also produce characteristic systemic *risks* for governance, many of which are mirror images of their benefits. First, HICs may amplify overlap and contestation, rather than producing order and coherence, where other institutions are closely aligned with treaties and FIGOs. Second, individual institutions may take on aspects of cooperation problems for which they are poorly suited, and diverse actions by multiple institutions may produce confusion, reducing substantive fit. Third, the “soft” institutions within HICs may reduce the focality and authority of incumbent treaties and FIGOs or weaken the incentives to establish new ones; here the institutional choice that produces political fit reduces the effectiveness of the governance system.

The full variety of HICs and their impacts on global governance cannot be encompassed within a single article. Our aim, then, is to initiate a research agenda that can more fully explore their structures, operations, and governance implications. Empirical research will be essential in this effort. While space limitations preclude us from engaging in extensive empirical investigation in this paper, we illustrate our arguments with diverse examples and set out analytical conjectures to facilitate the framing and testing of empirical propositions. In Sect. 5, we suggest fruitful avenues for future research.

## Introducing HICs

### Emergence of HICs

The explosion of diverse institutional forms, often within incumbent regime complexes, means that most issue areas today are governed by HICs. Consider the following examples.

The *climate change* HIC includes a range of institutions associated with the UN Framework Convention on Climate Change (UNFCCC), as well as other formal interstate institutions, including other treaties, international development banks and plurilateral “clubs,” many led by the United States (Keohane & Victor, 2011). It also encompasses IIGOs including the G7/8 and G20, multinational and regional environmental TGNs, and several transnational networks of subnational governments. In addition, PTROs set standards for private behavior (Abbott et al., 2016), and other private organizations engage in information sharing, operational activities and finance (Abbott, 2012). Finally, under the “voluntary commitment system” initiated by the UN Secretary-General and UNFCCC, numerous states, subnational governments, public–private partnerships, firms and civil society organizations have voluntarily pledged action. Thus, the climate change HIC is far more encompassing than either the “regime complex for climate change” (Keohane & Victor, 2011), which includes only international institutions, or the “transnational regime complex for climate change” (Abbott, 2012), which includes only transnational institutions.

The *global health* HIC (Hoffman et al., 2015) is centered on the World Health Organization (WHO), complemented by its regional organizations (e.g., Pan-American Health Organization) and other regional institutions. FIGOs including the World Bank, United Nations Development Program and UNICEF also address aspects of health. In addition, global health governance is carried out by global and regional TGNs, which harmonize and strengthen regulation of pharmaceuticals, medical devices, food, blood and other products, and facilitate cooperation on inspections. TPPPs and multi-stakeholder institutions play prominent roles in developing and delivering medicines, vaccines and health care; important examples include GAVI – the Vaccine Alliance (GAVI), the Coalition for Epidemic Preparedness Innovations (CEPI), and the Roll Back Malaria Partnership. The Global Fund to Fight AIDS, TB and Malaria is a unique multi-stakeholder institution focused on financing. Finally, civil society and professional organizations directly provide health services (e.g., Médecins Sans Frontières), respond to disease outbreaks (e.g., Global Outbreak Alert and Response Network [GOARN]), and engage in research, advocacy and funding. The Bill & Melinda Gates Foundation now plays a central role, shaping the agenda for global health governance (Hanrieder, 2015) and supporting development and deployment of new health technologies.

The HIC for the *prudential regulation of financial institutions* (Black, 2013) includes FIGOs such as the IMF, World Bank and OECD, but governance is centered on a series of prominent TGNs, which govern banking (Bank for International Settlements, Basel Committee on Banking Supervision, International Association of Deposit Insurers), securities (International Organization of Securities Commissions), insurance (International Association of Insurance Supervisors), auditing (International Forum of Independent Audit Regulators), and other issues. The private International Accounting Standards Board prescribes relevant accounting rules. The G20, an IIGO, provides overall guidance; it sponsored the creation of the Financial Stability Board (FSB) (a TGN) to coordinate the complex (Rixen & Viola, 2020).

The *cyberspace* HIC (Nye, 2014) includes UN bodies and other FIGOs that deal with core issues such as telecommunications and intellectual property, as well as emerging issues in areas including human rights, the “information society” and development. It also includes IIGOs such as the G7/8, G20 and 3G, plus TGNs addressing data protection and privacy, intelligence sharing and law enforcement. Private organizations such as the Institute of Electrical and Electronics Engineers (IEEE) adopt technical standards for internet and communications technologies. Most importantly, core cyberspace policy standards are promulgated by a diverse set of private, public–private and multi-stakeholder institutions, including the Internet Corporation for Assigned Names and Numbers (ICANN), the Internet Engineering Task Force, and the World Wide Web Consortium (W3C).

The *nuclear safety* HIC (Alger, 2008) includes several multilateral treaties, including the Convention on Nuclear Safety; the International Atomic Energy Agency (a FIGO with many subsidiary bodies and soft law norms) and other FIGOs; the Nuclear Safety and Security Group, a technical network sponsored by the G8; multilateral and regional TGNs of nuclear regulators; transnational scientific bodies; the World Nuclear University, a TPPP; and private industry associations.

### The HIC concept as analytical lens

In this subsection we introduce four characteristics that define the HIC concept in relation to that of the regime complex.

*First*, a HIC is characterized by a relatively high degree of *institutional diversity*. As the examples above demonstrate, HICs include a wide range of interstate (formal and informal), infra-state, public–private, and private institutional forms, with significant differences in membership, legal and political authority, modes of operation and other important features, discussed further below. Regime complexes, in contrast, are understood to be composed of relatively homogeneous institutions – denoted simply as “elemental regimes” (Raustiala & Victor, 2004: 279) or “elemental institutions” (Alter & Raustiala, 2018: 332). In the literature, these are all interstate institutions – treaties and treaty-based FIGOs.

Because both HICs and regime complexes are types of global governance complexes (Eilstrup-Sangiovanni & Westerwinter, 2020), we can array them both along a continuum of institutional diversity. Regime complexes, with relatively little institutional diversity, cluster near the low end, the endpoint being a complex comprising only two FIGOs or treaties identical in form. In fact, however, regime complexes can include both treaties and FIGOs, each of those forms can exhibit considerable variation, and their numbers and proportions can also vary. While we treat them separately for purposes of comparison, then, one can view even pure regime complexes as low-diversity HICs, and can conceptualize the entire continuum as depicting the range of HICs.^7^

HICs with relatively greater institutional diversity occupy the remainder of the continuum. These too vary widely in the types, numbers and proportions of component institutional forms (for empirical examples, see Pattberg & Widerberg, 2020). A HIC located near the regime complex region – e.g., one comprising multiple FIGOs plus a TPPP – would satisfy our definition, but would be unlikely to exhibit strongly the characteristic features of HICs. As one moves toward the high end of the continuum, however – typified by the climate change and global health HICs – institutional diversity increases significantly, and we expect those complexes to exhibit more strongly the characteristic features of HICs.

Second, the components of a HIC are *global governance institutions*. All perform one or more governance functions, such as standard-setting, monitoring, enforcement, financing of implementation, or producing and disseminating information.^8^ We do not treat members (e.g., states, agencies, firms or NGOs) or stakeholders of institutions as part of the complex, and do not include organizations that merely advocate policies or “provide input and feedback” to governance institutions (Henning & Pratt, 2020), although those groups certainly influence governance indirectly.

Third, the institutions within a HIC address a *common set of governance problems*, revolving around, for instance, climate change, nuclear safety or prudential regulation of financial institutions. Specifying these problems defines the *substantive* boundaries of a complex, although such specifications are inherently imprecise. The regime complex literature generally specifies an “issue area” (e.g., Henning & Pratt, 2020; Raustiala & Victor, 2004),^9^ but issue areas are unobservable analytical categories, and scholars define them differently depending on their research questions.^10^ Further, a HIC may address problems that are narrower or broader than an identified issue area, or that intersect multiple issue areas.

Scholars can likewise define the *institutional* boundaries of governance complexes in line with their research questions. For example, even though a HIC includes multiple institutional forms, scholars may appropriately focus only on legalized interstate institutions to address conflict between binding rules (Raustiala & Victor, 2004); only on interstate institutions to address cross-institutional strategies of states (Alter & Meunier, 2009); or only on transnational institutions to highlight their exclusion from the regime complex literature (Abbott, 2012).^11^

In many settings, however, it is important to consider the HIC as a whole, using the broader analytical lens developed in this paper. A broader lens is clearly necessary where research questions encompass multiple institutional forms; it is even necessary where they focus only on particular institutional forms, if the presence of other forms may affect their actions or interactions, e.g., where the actions of informal, infra-state or private institutions influence the operations or impacts of treaties or FIGOs. More broadly, it is necessary whenever scholars make *general* analytical claims about global governance in domains governed by institutionally diverse HICs.

Fourth, a HIC is a “*complex*,” not just a collection of institutions. The component institutions of a HIC address a common set of problems on a continuing basis; all have appropriate authorities and capabilities to govern in that domain. As a result, those institutions interact with one another on a continuing basis: they take account of one another’s actions; influence one another’s normative development and governance effectiveness (Gehring & Oberthür, 2009); and generate other interactional effects.^12^ Such interactions and effects are systemic features of HICs, which constitute them as governance complexes. Thus, only institutions involved in such interactions are part of the complex.

Many of the characteristic features, benefits and weaknesses we identify here reflect the effects of interactions among component institutions. For example, interactions based on functional differentiation may generate a division of labor, which can increase the effectiveness of the governance system through comparative advantage and mutual reinforcement. And interactions based on informal hierarchy can render the system more ordered and coherent. On the other hand, institutional interactions may weaken incumbent institutions, reducing system effectiveness, or amplify competition and conflict, reducing system coherence. Because of these effects, a HIC is not simply a group of “parallel” (Alter & Meunier, 2009) or “fragmented” (Keohane & Victor, 2011) institutions, but a governance system (Faude & Gehring, 2017; Gehring & Faude, 2013).

This does not mean that all component institutions necessarily share common norms or epistemes, or that there is no conflict among them. Institutions may have epistemic or normative differences, frequently reflecting those of their members, producing disagreement over the best ways to address governance problems. Where differences arise out of contending spheres of authority, with different views of the governance goal or common good, conflict will be especially intense (Kreuder-Sonnen & Zürn, 2020). In the cyberspace HIC, for example, some institutions (led by the US and other Western states) favor an open internet governed by multi-stakeholder institutions, while others (led by China and Russia) view an open internet as dangerous and promote strict regulation by states (Flonk et al., 2020).

### Structural features of HICs

In this subsection we first introduce the characteristic structural features of HICs in relation to the well-established features of regime complexes. We then develop conjectures as to how those features affect the governance outcomes highlighted by the regime complex literature.

The literature identifies two characteristic structural features of regime complexes, both of which reflect their relatively homogeneous institutional composition.

- First, component institutions “*overlap*” in terms of both authority and state membership; as a result, multiple institutions claim authority to govern particular issues, and their common member states are all subject to their (potentially inconsistent) rules.
- Second, there is *no formal hierarchy* among overlapping component institutions. This is “the key political feature of a regime complex—the feature that drives the critical dynamics and strategic interactions that characterize politics within a regime complex” (Alter & Raustiala, 2018: 332; see also Raustiala & Victor, 2004: 279).

Institutionally diverse HICs differ from regime complexes in terms of both overlap and hierarchy. HICs exhibit relatively greater functional differentiation, and thus less overlap; while they too typically lack formal hierarchy, they exhibit greater informal hierarchy. As a result, they produce different dynamics, interactions, systemic effects and outcomes.

Importantly, we think of functional differentiation and hierarchy not as fixed values, but as variable dimensions along which HICs and regime complexes can vary (compare Henning & Pratt, 2020). Both dimensions derive from institutional diversity. As with the underlying continuum of institutional diversity, then, we expect HICs to assume values on both dimensions that are systematically higher, on average*,* than those of regime complexes.

#### Functional differentiation

In contrast to the treaties and FIGOs of regime complexes, the institutional components of HICs are substantially more differentiated in membership and authority, the two determinants of “overlap.” For example, only FIGOs, treaty bodies and IIGOs have state members and can adopt standards that authoritatively address states; among those, only treaty bodies and a few FIGOs can adopt legally binding rules. With rare exceptions, TGN standards address only participating government agencies, while TPPP and private standards address only private actors; none is legally binding.

Alternative institutional forms also differ from interstate institutions, and from one another, in their governance functions and techniques. For example, TGNs typically focus on harmonizing regulatory standards and collaborating on implementation, e.g., inspections and enforcement. Subnational government associations typically focus on mutual learning in areas within their members’ jurisdiction, while TPPPs often focus on operational activities that draw on the resources and capabilities of public and private actors.

As a result of this differentiation, HICs face relatively fewer conflicting authority claims and less challenging problems of overlap – including rule inconsistency, instability and conflict – than do regime complexes. That said, if a HIC includes multiple FIGOs or treaties, nothing prevents them from asserting overlapping authority claims; the same may be true of other authoritative institutions of a single form, such as TGNs. But the presence of additional institutions of different forms does not proportionally increase the likelihood of authority conflicts.

The global health complex illustrates this effect. WHO remains the focal institution, especially on norm-setting and infectious disease response, although its focality has been reduced in many domains (Hanrieder, 2015). During the Covid-19 pandemic, WHO has focused on declaring an outbreak of international concern to trigger member state obligations, approving vaccines for emergency use by UN agencies and governments, and communicating technical guidance to states. Over time, WHO has encountered significant authority conflicts with other FIGOs, including the World Bank, UNICEF, WTO and UNAIDS (a joint UN program involving several FIGOs). More recently, it has also faced modest overlaps with other institutional forms, notably with the Gates Foundation on health financing; the Global Fund, a TPPP, also provides substantial financing, but only on three diseases.

Overall, however, the diverse institutional forms in the complex deal with different actors, exercise different forms of authority, and perform different governance tasks, with limited overlap and conflict. IIGOs (e.g., G20) coordinate national responses; TGNs strengthen pharmaceutical regulation. TPPPs (e.g., GAVI, CEPI) finance and deliver medicines and vaccines. Civil society organizations provide health services (e.g., Médecins Sans Frontières), respond to outbreaks (e.g., GOARN), and provide funding (e.g., Gates Foundation). WHO collaborates with all of these institutions to enlist their specific capabilities, as in the Access to COVID-19 Tools Accelerator and the COVAX vaccine initiative. While the pandemic response has been far from satisfactory, functional differentiation has almost certainly enhanced its effectiveness.

*As the diversity of their component institutional forms increases, HICs will be characterized by greater functional differentiation, and relatively fewer overlapping authority claims, than regime complexes composed of relatively homogeneous institutions.*

#### Informal hierarchy

While regime complexes and HICs both feature little *formal* hierarchy, the diverse component institutions of HICs exhibit greater *informal* hierarchy: “unofficial stratification … because of conscious or unconscious social processes,” as opposed to “official structures and rules allocating formal roles and positions at different levels” (Diefenbach & Sillince, 2011: 1516). While informal hierarchy also exists within regime complexes (Pratt, 2018), the institutional heterogeneity of HICs expands and reinforces it.

The literature on informal hierarchy in world politics focuses primarily on relations among states, not institutions (e.g., Lake, 1996; McConaughey et al., 2018; Stone, 2011; compare Mattern & Zarakol, 2016). We therefore draw on Pratt (2018), which analyzes why certain FIGOs in a regime complex may defer to others, “generat[ing] an informal hierarchy of regulatory bodies…” (Pratt, 2018: 587).^13^

We build on the two distinct mechanisms that Pratt develops to explain inter-institutional deference. First, “functional efficiency” leads institutions to defer to others with greater expertise, more effective decision-making, stronger authority for the problem at hand, or other complementarities. This reduces “inefficient overlap and inconsistencies.” Second, “member state power” leads institutions to defer to others whose member states are relatively stronger. This results in “power-based hierarchy” among institutions (Pratt, 2018: 580). We consider both mechanisms below, arguing that both apply more forcefully in HICs than in regime complexes because of HICs’ greater institutional diversity.^14^

Overall, we conjecture that:

*As their institutional diversity increases, HICs will feature greater informal hierarchy among component institutional forms than will relatively homogeneous regime complexes.*

#### Functional efficiency

Formal interstate institutions are highly institutionalized, allowing for the centralization of cooperative activities and institutional independence (Abbott & Snidal, 1998). Treaties and (some) FIGOs alone can create legally-binding rules for states and implement monitoring and enforcement procedures (although these are often weak in practice). Member states can implement agreed rules domestically through binding law. State membership also provides democratic legitimacy, enabling formal interstate institutions to adopt principles, norms and goals that diverse actors, not simply states, see as authoritative.

The functional efficiency rationale therefore leads us to conjecture that:

*Informal, infra-state, public–private and private institutions within HICs will tend to defer to legal rules, norms and other commitments adopted by formal interstate institutions*.

Informal and transnational institutions, such as IIGOs, TGNs and TPPPs, are less highly institutionalized and cannot adopt legally binding rules; hence they cannot perform many of the governance functions of treaties and FIGOs. However, they can effectively perform functions such as adopting coordination standards, disseminating information, and building trust; they have relatively low formation and operating costs, and so are more malleable and flexible (Abbott & Faude, 2020).

Each of these institutional forms also has unique strengths. TGNs, for example, derive authority and legitimacy from the technical expertise and domestic authority of member agencies. TGNs can make decisions effectively because of participants’ common knowledge and epistemic orientation; can build trust among officials; and can implement decisions through binding domestic regulations. They are particularly valuable in addressing complex, technical policy issues. TPPPs and private institutions draw on the material and subjective resources and capabilities of non-state actors, and can effectively address problems in which such actors are centrally involved.

Functional efficiency therefore leads us to conjecture that:

*Formal interstate institutions will tend to defer to informal and transnational institutions on the types of cooperation problems and governance tasks which those institutions can address effectively at lower cost.*

The climate change HIC illustrates both forms of deference. Numerous private, public–private and sub-state organizations defer to the goals, norms and approaches of the UNFCCC. Most notably, while multiple private carbon offset schemes expand on the UNFCCC’s Clean Development Mechanism (CDM), most explicitly recognize CDM rules, applying them to private targets through their own voluntary mechanisms (Green, 2013). Similarly, many public, public–private and private organizations participating in the voluntary commitment system explicitly follow UNFCCC priorities, addressing recommended “action areas” and adopting recommended cooperative structures.

At the same time, the UNFCCC, as well as the World Bank, UNEP and other FIGOs, defer to non-state organizations on activities that centrally involve private actors, including norms for voluntary climate offsets, pledges of action on mitigation, “green bonds,” standards for corporate environmental reporting, private financing, and renewable energy projects (Abbott et al., 2016). Similar examples can be observed in other fields. In labor governance, for instance, private institutions such as the Fair Labor Association (FLA) incorporate ILO standards into their voluntary codes of conduct. While the ILO adopted a Tripartite Declaration of Principles concerning Multinational Enterprises and Social Policy in 1977, a rare step for a FIGO, it largely defers to private institutions for voluntary business standards (Marks & Wouters, 2017).

#### Power

Interstate institutions draw on member states’ authority to exercise hierarchical power domestically, by enacting and enforcing national laws. TGNs, similarly, draw on the authority of member agencies to adopt and enforce binding domestic regulations, typically pursuant to state enactments. Even the shadow of hierarchical state power is often sufficient to induce sub-state, public–private and private actors to defer to the demands of interstate institutions. Indeed, private actors often strive to anticipate future exercises of state (and interstate) power and shape their behavior accordingly, to avoid subsequent (non-)compliance costs (Green, 2014).

However, institutions composed of powerful member states or agencies, and institutions to which states have delegated extensive authority, cast stronger shadows of hierarchy than weaker institutions (Pratt, 2018). The G20, for example, represents sufficient state authority and power to coordinate all the TGNs in the financial HIC. WHO, in contrast, has universal membership, diluting state power, and only limited delegated authority and resources. While other institutions defer to its expertise and legitimacy, the Global Fund, GAVI, Médecins Sans Frontières, and other institutions need have little concern for its power.

Conversely, some sub-state, private and public–private institutions have substantial authority and power due to their membership, resources, expertise or legitimacy; they will grant less deference to inter-state institutions and receive more themselves. Business-dominated PTROs and the Gates Foundation, which has far greater financial resources than WHO, are cases in point.

The member power rationale therefore leads us to conjecture that:

*Infra-state and non-state institutions will tend to defer to the rules of interstate institutions, especially those with powerful state members.*

*Sub-state and non-state institutions will tend to defer to the rules of TGNs, especially those with agency members from powerful states.*

In each case, the actual degree of deference will depend on the relative authority and power of the specific institutions involved.

## Conflict within HICs

Thus far we have emphasized how the institutional diversity of HICs reduces overlapping authority claims and promotes ordering through informal hierarchy. HICs can, however, experience inter-institutional discord and conflict that reduce governance effectiveness. Yet even here their characteristic structural features tend to ameliorate conflict.

First, as already discussed, *overlap* in authority claims and membership – the main source of discord in regime complexes – remains possible in HICs between authoritative institutions of the same or similar type, such as treaties and FIGOs (compare Drezner, 2013). However, overlap is reduced across different institutional forms, and to the extent other component institutions take on governance tasks that would otherwise have fallen to treaties and FIGOs. Similarly, the presence of other institutional forms may reduce the gradual expansion of authority by interstate institutions – “mission creep” – which can generate overlap over time.^15^

Second, powerful states and other powerful constituent actors, such as business firms, may engage in cross-institutional strategies to *bypass* opponents, veto players or burdensome procedures in incumbent institutions; to seek less burdensome forms of cooperation; or to pursue other individual goals (Alter & Meunier, 2009; Manulak & Snidal, 2020; Vabulas & Snidal, 2013). The multiplicity of institutions in HICs allows actors some latitude to “forum shop,” shift issues to more favorable forums, and create new institutions that favor them.

Third and most conflictual, powerful states and other powerful actors may create new institutions to directly *contest* “the rules, practices or missions of” incumbent institutions, a process known as “contested multilateralism” (Morse & Keohane, 2014).

The United States frequently uses strategies of bypassing and contestation. In the security domain, for example, the US created the Proliferation Security Initiative, Australia Group and Missile Technology Control Regime, all with narrow memberships of like-minded states: “coalitions of the willing” (Eilstrup-Sangiovanni, 2009). On climate change, the US created the Major Emitters Forum and other informal “clubs” of states that resisted strong UNFCCC emissions limits (Keohane & Victor, 2011). Other industrialized states also pursue these strategies, and changes in the global power distribution have accelerated the trend, with China recently following suit (Vabulas & Snidal, 2020).

Non-state actors likewise pursue strategies of bypassing and contestation. Business firms are notorious for establishing business-dominated PTROs to compete with civil society-sponsored institutions such as the Forest Stewardship Council (FSC), using the “second mover” advantage their resources and capabilities give them (Abbott & Snidal, 2009). And GAVI was created through the efforts of the Gates Foundation, which sought to bypass WHO to develop, finance and deliver vaccines more efficiently.

These strategies are responsible for some of the institutional proliferation in HICs, and contribute to discord and conflict within them. However, bypassing appears far more common than direct contestation.^16^ For example, the US created the Australia Group to facilitate technical expert consultations infeasible within the chemical and biological weapons conventions (Vabulas & Snidal, 2013); it created the G20 to encourage financial reform without the burdensome procedures of the IMF (Viola, 2015:27). Neither was intended to undercut the incumbent institution. While WHO may view GAVI as a competitor – in part because of epistemic differences with the Gates Foundation – Gates created GAVI primarily to fill perceived operational and financial gaps, not to contest WHO’s legitimacy.

Bypassing is more benign than contestation: it may reinforce epistemic or normative disagreements, but less often leads to outright conflict. Contestation and conflict occur primarily when states act out of dissatisfaction with the *regulatory rules* of incumbent institutions and seek to change them; they are less likely when states act out of dissatisfaction with the decision-making *procedures* or *effectiveness* of incumbent institutions and seek merely to avoid or enhance them (Faude & Fuß, 2020).

Bypassing strategies can even benefit cooperation (Faude, 2020a). They provide “systematic pathways through or beyond gridlock” (Hale & Held, 2017), and can facilitate inter-state compromise, inducing cooperation on substance in return for less risky forms of institutionalization. A principal advantage of IIGOs is that they “allow states to bypass but not disrupt existing institutional arrangements – or even work alongside them” (Vabulas & Snidal, 2020: 42). IIGOs similarly “allow both established and rising power states to adapt institutions to changing power distributions without upsetting the prevailing order” (Id.: 41). Other institutions, such as TGNs, can alleviate gridlock in technical fields (Hale & Held, 2017).

While both bypassing and contestation remain possible, moreover, the structural features of HICs limit actors’ incentives and ability to pursue them. Importantly, HICs’ institutional diversity constrains choice among institutional forms through forum-shopping and institutional creation.^17^ Not all institutional forms are available to states, firms or other actors seeking to bypass or contest, and those that are may not provide the authority or capabilities they seek. In particular, few institutions are suitable for powerful states seeking to challenge incumbent treaties or FIGOs. An IIGO may provide the germ of a challenge – and most examples, like the Proliferation Security Initiative and Major Emitters Forum, involve IIGOs – but no other form can present a strong and immediate challenge. Even with bypassing, while states may generate modest benefits by shifting issues to IIGOs, TGNs or TPPPs, these will not substitute fully for treaties or FIGOs.

Informal hierarchy means that most alternative institutional forms – unless specifically designed for contestation or reflecting contending spheres of authority – are likely to defer to incumbent treaty-based institutions, aligning their norms and activities to benefit from the latter’s authority and legitimacy (Reinsberg & Westerwinter, 2021). For example, Green (2013) identifies a “coral reef” mechanism, in which “forms of public authority attract private actors,” which then adopt public rules as the basis for their own private standards (see also Green & Auld, 2017: 272–273). Such institutions supplement existing public rules, but rarely conflict with them.

Finally, institutional diversity can dampen some harmful impacts of bypassing and contestation. Even if states or other actors shift issues to different institutions or create new ones, doing so will not undercut the entire governance system: other institutions, created and managed by different actors, can to some extent take up the slack. Said another way, institutional diversity enhances *resilience,* both to exogenous shocks and to endogenous shocks created by cross-institutional strategies. Resilience is often thought to depend on institutional redundancy (Duit et al., 2010), and full redundancy is limited by institutional diversity. But the presence of diverse institutional forms that can address multiple aspects of a problem in different ways still provides significant resilience (Faude, 2020b).

## HICs and governance outcomes

In this section we consider other major implications of HICs’ institutional diversity, and the resulting systemic effects, for global governance outcomes. We first address HICs’ characteristic governance benefits, then their characteristic governance risks.

### Governance benefits

#### Substantive fit

“Fit” refers to congruence or compatibility between a governance system and the problems it seeks to address (Young, 2002). The institutional diversity of HICs – whose component institutions possess different forms of authority, address different targets, and utilize different governance techniques – enables them to offer superior “substantive fit” for many contemporary governance problems.

Contemporary global issues are often characterized by “problem diversity:” multiple sub-issues that present substantively different cooperation problems. For example, “climate change” involves at least four distinct inter-state cooperation problems, each with its own actors, incentives, constituencies and other attributes: (1) carbon emissions reductions, (2) financial transfers, (3) other responses including adaptation and geoengineering, and (4) scientific assessments (Keohane & Victor, 2011: 12–14).

Multifaceted problems require multifaceted institutional responses. This echoes the “law of requisite variety” from systems theory, which “suggests that for governance to be effective, the complexity of governance needs to match the complexity of the system being governed” (Ashby, 1962; see also Biermann et al., 2020).^18^ Because of their institutional diversity, HICs are better able than regime complexes to provide variegated governance responses: the component institutions of HICs can each address those aspects of a cooperation problem to which they are best-suited, potentially increasing the effectiveness of the system through comparative advantage and mutual reinforcement.^19^

One important variation among cooperation problems is their incentive structure or “problem type” (see Stein, 1982). Authoritative interstate institutions, especially treaties and FIGOs, are best-suited to addressing dilemmas of common interests, such as prisoner’s dilemma problems among states, where defection is a threat and credible commitments are essential.^20^ But alternative institutions can provide adequate or even superior responses to many other cooperation problems, frequently at lower cost. These include coordination problems and other dilemmas of common aversions, as well as problems characterized by uncertainty and dynamism (Abbott & Faude, 2020).

In addition, many contemporary global problems stem from the actions of sub-state and private actors as well as states, and require responses from those actors. Climate change, for example, becomes even more multifaceted when one considers that non-state actors contribute to problems such as emissions, financing and adaptation, and can contribute to effective governance responses (Ostrom, 2010). Where diverse actors contribute to problems and are necessary for effective responses, institutional complexity must match actor complexity. The alternative institutions within HICs are composed of different actors (e.g., government agencies, sub-national governments, business firms, NGOs); address those actor groups as targets; utilize governance techniques appropriate to those targets (e.g., voluntary standards); and contribute varied forms of expertise and other governance capacities.

In short, HICs can decompose the diverse facets of complex governance problems, addressing each through institutions with appropriate substantive fit. This important systemic effect can be seen in all the examples introduced above. On climate change, for example, the UNFCCC applies legally binding and non-binding rules and norms for states; IIGOs such as the G20 adopt broad principles and goals for the global community; FIGOs such as the World Bank provide financing and support national programs; TGNs such as the European Network of Heads of Environment Protection Agencies harmonize sectoral regulations and programs; TPPPs such as the Renewable Energy and Energy Efficiency Partnership (REEEP) experiment with operational projects; and PTROs such as the Verified Carbon Standard (VCS) supplement public rules with voluntary standards for business and other private actors.

In cyberspace, core functions such as maintaining the domain name and internet address systems are performed by a unique mix of institutions, including private technical bodies such as the Internet Engineering Task Force, and ICANN, previously state-controlled but recently privatized. Many of these institutions seek substantive fit by mimicking the network structure of the Internet in their decision-making processes. Treaty bodies implement rules for states on subjects including intellectual property, cybercrime and human rights. FIGOs such as the ITU, IIGOs including the G20, and other interstate bodies address policy issues in areas including development, telecommunications and the “information society.” The World Bank and other financial bodies support cyber development and policies in developing countries. TGNs harmonize regulations and enforcement in areas including telecommunications and data protection.

Institutional diversity also increases system effectiveness through mutual reinforcement. TGNs, TPPPs and PTROs (partially) fill governance gaps left by FIGOs and treaties, and extend FIGO/treaty rules to additional target groups (Green & Auld, 2017:272–273). Flexible, low-cost institutions can experiment with potential possible governance solutions (Hoffmann, 2011), and act as reservoirs of ideas that others can later adopt (Green & Auld, 2017:271). Finally, the diverse institutions within HICs simultaneously address problems in multiple ways, e.g., through legal rules for states plus voluntary standards for firms; or through broad goals for states, such as the Sustainable Development Goals, plus TPPP projects that generate learning on ways to achieve those goals.

In sum, we conjecture that:

*To the extent that cooperation problems are characterized by problem diversity, institutionally diverse HICs have a systematic substantive fit advantage over relatively homogeneous regime complexes.*

#### Political fit

“Political fit” refers to the incentive-compatibility of cooperative arrangements: the greater the political fit, the more a cooperative arrangement satisfies the preferences and demands of constituents and stakeholders, making governance more effective. The institutional diversity of HICs enables them to provide superior political fit for many contemporary governance problems, especially where constituents and stakeholders are themselves diverse.

Each component institution offers a specific “package” of governance benefits, limitations, costs and risks for its members, stakeholders and targets. This package reflects the institution’s unique authority, membership, substantive focus, modes of operation, and expertise. Within important limits, discussed below, a HIC provides diverse actors opportunities to participate in those institutions whose governance strengths, weaknesses, costs and risks most closely match their characteristics and preferences.

States have the broadest options: they can choose to address a governance problem through a treaty like the UNFCCC; a FIGO like WHO; an IIGO with limited membership like the G20; an informal club like the Australia Group; a multi-stakeholder institution like the Global Fund; or a TPPP like REEEP.^21^ States can also sponsor and support such institutions, from TGNs to PTROs, and states including the US and UK have actively done so. In addition, interstate institutions can sponsor and support other institutions (Manulak & Snidal, 2020).

Non-state actors have fewer options. They can, however, choose between participating in TPPPs or PTROs such as the FSC. They sometimes participate in TGNs, and have expanding opportunities to participate in FIGOs (Tallberg et al., 2013). Even where direct participation is not possible, actors can *support* institutions that fit their preferences; for example, business organizations supported the creation of the FSB, rather than a more powerful institution, to preserve governments’ ability to protect national financial industries. Regime complexes composed exclusively of formal interstate institutions cannot provide equally diverse opportunities.

Beyond providing distinct governance benefits and costs, each institutional form *empowers* particular actors, providing them direct roles in governance. For example, IIGOs empower executive officials (vis-à-vis legislatures), TGNs government agencies, and TPPPs and PTROs private actors. The prospect of empowerment incentivizes actors to create and participate in the institutional forms that benefit them, providing additional political fit.

In sum, we conjecture that:

*As the diversity of their component institutional forms increases, HICs will provide better political fit for diverse constituents and stakeholders than will relatively homogeneous regime complexes.*

#### Coordination and coherence

Effective coordination can enhance the governance effectiveness of HICs. For example, coordination mechanisms can encourage component institutions to address aspects of problems appropriate to their authority and capabilities. They can encourage institutions to take others into account, harmonize norms and policies, and learn from others’ experiences (see Zeitlin & Overdevest, 2020). These actions enhance substantive coherence, an important criterion for assessing the effectiveness of governance complexes (Keohane & Victor, 2011). Coordination mechanisms can facilitate inter-institutional collaboration, promoting more ambitious governance goals (Galaz et al., 2011). They can discourage the pursuit of damaging cross-institutional strategies and resolve conflicts.

We consider three distinct mechanisms of coordination:

- *institutional design for complementarity*, the path-dependent process (Raustiala & Victor, 2004:280) in which the founders of new institutions select institutional forms and designs to mesh with incumbent institutions (Reinsberg & Westerwinter, 2021; Westerwinter, forthcoming);
- *decentralized adaptation*, the primary mechanism in polycentric governance theory (Jordan et al., 2018; Ostrom et al., 1961), in which the managers of institutions adjust their governance activities to those of other institutions over time; and
- *strategic ordering*, in which actors (e.g., the US or UK) or institutions (e.g., WHO or G20) intentionally influence the creation, design or behavior of other institutions.

All three mechanisms are initiated by individual actors (design, strategic ordering) or institutions (adaptation, strategic ordering), not by HICs as such. However, the characteristic features of HICs are *conducive* to the effective use of all three mechanisms.^22^

First, functional differentiation and informal hierarchy encourage *contextual design*. Founders are well aware of the “existing global governance architecture” (Reinsberg & Westerwinter, 2021) and the capabilities and limitations of the institutional forms they choose to create. Consistent with functional differentiation, founders have incentives to structure institutional designs and mandates to avoid overlap with incumbent institutions^23^ – unless their goal is bypassing or contestation. Overlap increases transactions costs, invites norm conflicts and weakens cooperative focal points, creating instability. These costs are particularly high where incumbents have superior functional efficiency or greater power. In the latter case, incumbent institutions (or their members) may be able to exercise hierarchical control over new entrants (or their members), or otherwise impose significant costs. The founders of weak institutions will thus seek protected “niches” in which to operate (Abbott et al., 2016).

In addition, founders often design and mandate institutions to complement institutions of other forms, in what historical institutionalists call “layering” (Streeck & Thelen, 2005: 22–24; Fioretos, 2011, 389–391). For example, many TPPPs, such as REEEP, are created to develop practical experience in implementing treaty/FIGO standards, e.g., through demonstration projects (Andonova, 2010). Many PTROs, such as VCS and FLA, are designed to extend interstate rules to private targets through voluntary standards. Such institutions remain closely attuned to changes in their reference standards (Marks & Wouters, 2017:201), while the relevant FIGOs and treaty bodies learn from their activities.

Similarly, founders often create and design institutions to help fill governance gaps left by institutions of other forms. For example, FSC was created in direct response to the failure of treaty negotiations on sustainable forestry (Auld & Cashore, 2012). Gap-filling institutions select targets, substantive foci, norms and governance techniques with an eye to those gaps, and adjust their activities as other institutions narrow or widen them.^24^ The diverse institutional templates now available to founders enhance their ability to achieve complementarity, especially where incumbents are relatively homogeneous. The low formation costs, malleability and flexibility of alternative institutional forms allows founders and managers to take advantage of these options.

Second, for the same reasons, functional differentiation and informal hierarchy encourage *decentralized adaptation*. Institutional managers too have strong incentives to adjust designs, norms and activities to avoid costly overlaps, especially with functionally superior and more powerful competitors. Managers of weaker institutions may shift their mandates and activities into protected niches, or align them with stronger institutions, as in the “coral reef” mechanism noted above. The flexibility of alternative institutional forms increases their ability to adjust.

Finally, informal hierarchy is particularly conducive to *strategic ordering*. This coordination mechanism is a common feature of HICs:

- States, FIGOs and IIGOs sponsor TGNs to perform complementary governance tasks: the G20 sponsored the FSB to facilitate frank consultations among senior policymakers, supplementing the IMF (Clarke, 2014); the G8 sponsored the Nuclear Safety and Security Group to provide technical advice on safety standards, supplementing the IAEA (Alger, 2008); WHO sponsored the Blood Regulators Network to harmonize regulations over which it lacked direct authority. Influential IIGOs such as the G20 also steer TGN actions and set broad priorities that guide national officials, and thus the work of TGNs (Manulak & Snidal, 2020). Following the 2008 financial crisis, for example, the G20 steered financial TGNs toward coordinated efforts to address the systemic risks the crisis had exposed.
- Some FIGOs sponsor and support TPPPs, sub-state associations and PTROs to more effectively accomplish their own governance goals and enhance their influence. UNEP helped establish the Global Reporting Initiative (GRI) to promote corporate sustainability reporting; the World Bank created the Prototype Carbon Fund (PCF) to engage private actors in novel carbon emission reduction projects.
- Recently, states, FIGOs and treaty bodies have established governance “platforms” or “voluntary commitment systems” that institutionalize the sponsorship and steering of complementary institutional forms (van der Ven et al., 2017; Abbott, 2018; Orchestration, 2018). In climate change, for example, such platforms encourage the formation of TPPPs that elicit commitments to action by individual cities, firms and other actors. They establish criteria for the operations of those actors and institutions, and actively steer and support them in other ways.

Such interactions tend to produce tighter and more extensive inter-institutional linkages within HICs than are found in “loosely coupled” regime complexes (Keohane & Victor, 2011:8).^25^

The techniques of strategic ordering include meta-governance, orchestration and interplay management. In orchestration, for instance, an “orchestrator” (the steering actor or institution) engages other institutions as intermediaries, inducing them to act vis-à-vis their governance targets in ways that further the orchestrator’s goals (Abbott et al., 2015). Thus, UNEP sponsored, supported and steered GRI; the climate change governance platform encourages and steers non-state institutions. Orchestrators rely on inducements including persuasion; material assistance and ideational support; convening potential founders; shaping institutional agendas through information and norms; and endorsing institutions that meet their criteria.

Institutions with relatively high hierarchical status are best positioned to utilize all of these techniques.^26^ Incumbent treaties and FIGOs, such as the UNFCCC and WHO, are often “focal” in their issue areas (Jupille et al., 2013); other institutions turn to them for authoritative norms, information and guidance. Powerful states and prominent institutions of other forms, such as the G20 and FSB, also possess substantial focality. Where an institution receives deference because of its expertise, decision-making procedures, authority or legitimacy, other institutions will likewise be responsive to its norms, guidance, ideational support and endorsement. Where an institution’s power is responsible for deference, other institutions will be responsive to its persuasion, carried out in the “shadow” of its power.

We therefore conjecture that:

*As the diversity of their component institutional forms increases, functional differentiation and informal hierarchy will make HICs increasingly conducive to coordination mechanisms such as contextual design, decentralized adaptation and strategic ordering.*

*Coordination mechanisms will produce relatively tight coupling among the component institutions of HICs, and thus relatively strong coherence in rules, policies and programs*

### Governance risks

#### Overlap and conflict

Where infra-state, public–private and private institutions align their norms and programs with particular treaties or FIGOs – or where FIGOs or powerful member states orchestrate or manage those institutions – HICs may amplify problems of overlap and conflict rather than muting them. For instance, if an FIGO expands its authority into an issue area governed by an incumbent FIGO, overlap and conflict may ensue, creating costs for member states. But if TGNs, TPPPs or PTROs aligned with the expansionary FIGO expand their authority in parallel, they too may overlap institutions aligned with the incumbent, extending conflict throughout the HIC system, hampering coordination and creating costs for multiple actor groups.

During the 1980s, for example, the World Bank began to finance developing country health programs, creating conflict between the Bank (with a neoliberal focus on efficiency and reducing public expenditures) and WHO (with a focus on social determinants of health and national health systems) (Lee, 2009: 111–115). Private foundations, TPPPs with business participation, health and development NGOs, and other institutions closely aligned with or orchestrated by one or the other institution would also have been pulled into the conflict, expanding the costs.

The US and other powerful states use IIGOs or TGNs to bypass opponents and veto players in incumbent treaties or FIGOs, or to contest existing rules. Again, if alternative institutions are aligned with or orchestrated by the incumbent or the challenging institution (or their dominant member states), they too will be pulled into the conflict, undermining substantive fit and coherence. More broadly, we expect unequal power and power-based strategies to be as common in HICs as in regime complexes. Powerful states in treaty bodies, FIGOs and IGOs; regulatory agencies from powerful states in TGNs; and powerful business firms, foundations and NGOs in TPPPs and PTROs all utilize their power to further their own ends.

#### Substantive fit

Functional differentiation allows the component institutions of HICs to address those aspects of cooperation problems for which they are best suited. Without sufficient coordination, however, individual institutions may take on aspects for which they are poorly suited, reducing substantive fit. Such misalignments could result from simple misjudgment, from the excessive ambition of founders and managers, or from resource competition among them.

Multiple institutions that address problems in mutually reinforcing ways enhance system effectiveness. But multiplicity can also create confusion: overlapping treaty, FIGO or TGN rules may lead to inconsistencies, while diverse voluntary standards require producers to choose the most beneficial standards and consumers to choose the most appropriate certified products, often with inadequate information. In addition, multiple institutions may insert themselves into areas characterized by network externalities, where governance by only a single institution is desirable (compare Lipscy, 2015).

#### Political fit

While HICs constrain cross-institutional strategies, they still provide actors some leeway to join or create institutions that impose relatively weak obligations (Benvenisti & Downs, 2007; Drezner, 2013; Hale et al., 2013). In addition, while IIGOs, TGNs, TPPPs and PTROs rarely compete directly with treaty-based institutions, they do provide alternatives for states and other actors demanding governance. As these institutions proliferate, they reduce the focality of incumbent treaties and FIGOs, individually and as a class, reducing the scope of their authority. Even though GAVI and the Gates Foundation were not designed to contest WHO’s legitimacy, for example, they have reduced its focality (Hanrieder, 2015).

Similarly, where “soft” institutional forms, such as IIGOs and PTROs, address problems, it may be more difficult for treaty bodies or FIGOs to generate the political will to take “hard” action on those problems – even if hard action would be superior, as with dilemmas of common interests (Lake, 2020; see Stein, 1982).^27^ This argument applies even where soft governance (partially) fills gaps left by stronger institutions. Business firms and other actors may thus use their resources, managerial capacity and other capabilities to create TPPPs and PTROs based on self-regulation and market-oriented approaches, intentionally aiming to blunt political pressure for stronger regulation (Abbott & Snidal, 2009). For all these reasons, HICs risk combining the benefits of multiplicity and diversity with relatively weak forms of governance.

## Conclusion

As alternative institutional forms have proliferated within incumbent regime complexes, HICs have become the most common form of global governance complex. Our first contribution, then, is to introduce and develop the HIC concept as a descriptive category and analytical lens.^28^ Crucially, HICs comprise diverse institutional forms: formal and informal; interstate, infra-state, public–private and private. The HIC concept thus captures, empirically and conceptually, governance institutions and institutional interactions overlooked by literatures focusing exclusively on more homogeneous (typically formal interstate) institutions.

The lens of regime complex theory retains analytical power in settings governed only by formal interstate institutions, or by HICs predominantly composed of such institutions, near the low end of the continuum of institutional diversity. The regime complex lens is also valuable for research questions that solely involve such institutions and their interactions – so long as the presence of other institutional forms does not significantly affect them. The HIC lens, in contrast, is fruitful in areas governed by diverse institutional forms, for research questions involving interactions among disparate institutions, and for making general claims about governance in issue areas occupied by HICs.

Our second contribution is to analyze the impact of HICs on the effectiveness of global governance. The structural features of HICs modify two central implications of regime complex theory: their functional differentiation limits overlapping claims to authority, and their informal hierarchy produces greater ordering and complementarity. HICs may still suffer from epistemic and normative differences, cross-institutional strategies and contestation, but their structure often limits these sources of conflict.

The institutional diversity of HICs also creates other characteristic governance benefits – including substantive fit for multifaceted cooperation problems and political fit for diverse stakeholders. HICs are conducive to mechanisms of coordination, which can enhance coherence. Yet HICs also create characteristic governance risks. Institutional diversity and multiplicity may lead to confusion; inter-institutional alignments may amplify problems of overlap, discord and conflict; and the predominance of “soft” institutions may weaken or forestall the creation of stronger institutions, creating a complex that is diverse and resilient, but dominated by shallow forms of cooperation.

We noted at the outset that our goal is to initiate a research agenda on HICs, including far more empirical research than we can incorporate here. That agenda is a rich one.

- First, scholars can continue to document the makeup, structure and operation of HICs in diverse issue areas.
- Second, the institutional composition of HICs varies widely along the continuum of institutional diversity: HICs include different types, proportions and numbers of institutional forms. Scholars can document such variations, analyze the causal factors responsible for them, and consider their impact on governance effectiveness.
- Third, scholars can analyze how cross-institutional strategies for individual gain, strategies of bypassing procedural deficiencies and veto players in incumbent institutions, and strategies of direct contestation operate in HICs. It is important to document the use and impacts of such strategies empirically, and to explore the conditions under which the structure of HICs constrains or encourages them.
- Fourth, we have identified three mechanisms of coordination that can enhance coherence within HICs. Scholars can explore how actors and institutions apply these mechanisms, and analyze the conditions under which they are effective.
- Finally, we have identified a number of analytical conjectures, which can be operationalized as empirically testable propositions in terms appropriate to specific research questions and issue areas. Additional conjectures are of course possible: for example, our analysis might suggest that HICs are more likely to form in areas where incumbent treaties and FIGOs face deadlock; and that the tendency of weaker institutions to align their norms and activities with those of stronger institutions will reduce experimentation and renders HICs resistant to change.

In all these areas, the crucial first step is to accept the growing importance of diverse governance institutions and the hybrid institutional complexes they constitute, moving beyond the study of formal interstate institutions and regime complexes.

## Footnotes

1 Other illustrations include the global governance of energy (Downie, 2020), artificial intelligence (Cihon et al., 2020), and forestry (Zeitlin & Overdevest, 2020).

2 For this reason, it is rarely feasible to compare HICs to regime complexes in the same issue areas.

3 On the prevalence of regime complexes, see Alter and Raustiala (2018). Regime complexes arose in large part because treaties and FIGOs proliferated during the second part of the twentieth century (Shanks et al., 1996), leading to concerns over “treaty congestion” (Brown, 1992: 679) and “regime density” (Young, 1996: 20).

4 A governance “architecture” is an overarching view of governance arrangements at a point in time, including actors, institutions, ideas, principles and norms (Biermann & Kim 2020: 4–7). Thus, HICs and their component institutions and constituent actors are elements of the current governance architecture, but not equivalent to it.

5 Growth rates are not equal, and different forms are more numerous, in different issue areas (Westerwinter et al., 2021). Theoretically, institutional proliferation should be more prevalent in issue areas where network effects are low, so that a single focal institution is not essential, and where barriers to entry are low (Lipscy, 2015). Most alternative institutions have relatively low costs, however, lowering entry barriers.

6 For early steps in this direction, see; Betsill et al. 2015; Bulkeley et al. 2014; Green 2013; Green and Auld 2017; Gómez-Mera 2017; Widerberg et al. 2016.

7 Regime complexes also vary in their degree of integration (Keohane & Victor, 2011) and other systemic characteristics (Henning & Pratt, 2020).

8 While some scholars consider only rule-making institutions (e.g., Abbott et al., 2016; Green & Auld, 2017), we incorporate additional governance functions (see also Keohane & Victor, 2011, which incorporates financing and environmental assessment institutions).

9 Henning and Pratt (2020) usefully define an issue area in terms of substantive “spillovers” among individual issues.

10 For example, the plant genetic resources regime complex (Raustiala & Victor, 2004) addresses a subset of problems within the biodiversity regime complex (Morin et al., 2017). Similarly, Keohane and Victor (2011:12–14) see “climate change” as composed of multiple sets of problems, from mitigation to financial transfers; a HIC could address any one of these.

11 If only an interstate regime complex is active in a domain, of course, one must focus only on interstate institutions.

12 To emphasize institutional interactions, one can model HICs as networks, “structures that emerge via … patterned interactions [among actors] as individuals select who they interact with” (Alter & Raustiala, 2018).

13 Pratt operationalizes deference as one FIGO adopting or recognizing rules promulgated by another, or changing its practices to support another’s rules. The literature also considers other bases for hierarchy, including dominance and legitimacy (Mattern & Zarakol, 2016).

14 The “member state power” rationale does not apply directly to non-interstate institutions, but we extrapolate power considerations to other institutional forms.

15 A focus on common problems also makes competition for funding, authority and other resources likely (Abbott et al., 2016).

16 Domestic considerations drive some instances of institutional creation, producing relatively little international conflict. For example, policy-makers may face domestic political polarization that makes cooperation through treaties and FIGOs impossible; independent regulatory agencies may create TGNs to reinforce their domestic autonomy and expert authority (Roger, 2020).

17 The extent of “outside options” for states or other actors determines their ability to threaten exit from institutions – shifting issues to other institutions or forming new ones – to obtain distributional benefits (Lipscy, 2015). While HICs appear to present numerous institutional options, in fact relatively few are meaningfully available. Cross-institutional strategies of contestation remain possible within each institutional form.

18 It is, however, inefficient for governance to be significantly more complicated than the system being governed.

19 The “gridlock argument” attributes deadlock in international institutions in part to problem diversity (Hale et al., 2013). By considering only formal interstate institutions, however, this argument underestimates the potential of HICs to facilitate cooperation (see Hale & Held, 2017).

20 IIGOs and TGNs may also contribute to addressing such problems where incentives to defect are relatively weak.

21 While under certain conditions states have incentives to assign new cooperation problems to incumbent (frequently interstate) institutions (Jupille et al., 2013), often they instead assign problems to alternative institutions, based on their governance benefits and low costs (Abbott & Faude, 2020).

22 To be sure, if a HIC faces discord and conflict among component institutions it will be difficult for these coordination mechanisms to operate effectively.

23 Westerwinter (2020), for instance, shows that IGOs are more likely to join TPPPs when the latter perform governance functions substantially different from those provided by IGOs.

24 While alternative institutions such as PTROs are rarely perfect substitutes for treaty or FIGO rules, deadlock in a focal incumbent institution may leave few alternatives.

25 On linkages, see Pattberg et al. 2018; Betsill et al. 2015.

26 Kahler (2020) suggests that institutional diversity and informal hierarchy are a beneficial combination, allowing states and FIGOs to manage non-state institutions through orchestration.

27 The counterfactual is difficult to assess, however: given deadlock and cumbersome decision-making processes, treaties or FIGOs might well not act even without preemptive action by business.

28 This contributes to the nascent literature on complex global governance (Kahler, 2016; Lake, 2020; Ruggie, 2014).

## References

[CR1] 2018. Orchestration: Strategic Ordering in Polycentric Climate Governance. In Andrew Jordan, Dave Huitema, Harro van Asselt, Jonas Schoenefeld and Johanna Forster, eds., *Governing Climate Change: Polycentricity in Action?* Cambridge University Press.

[CR2] Abbott KW (2012). The Transnational Regime Complex for Climate Change. Government and Policy: Environment and Planning C.

[CR3] Abbott, K. W., & Benjamin, F. (2020). Choosing Low-Cost Institutions in Global Governance. *International Theory*, published online, 11 June.

[CR4] Abbott KW, Snidal D (2009). Strengthening International Regulation through Transnational New Governance: Overcoming the Orchestration Deficit. Vanderbilt Journal of Transnational Law.

[CR5] Abbott KW, Green JF, Keohane RO (2016). Organizational Ecology and Institutional Change in Global Governance. International Organization.

[CR6] Abbott, K. W., Philipp G, Duncan S, & Bernhard, Z. (eds.) (2015). *International Organizations as Orchestrators*. Cambridge.

[CR7] Abbott, K.W., Kauffmann, C., & Jeong, RL. (2018). The Contribution of Trans-Governmental Networks of Regulators to International Cooperation. OECD Regulatory Working Papers, No. 10. Paris: OECD Publishing.

[CR8] Alger, J. (2008). *A Guide to Global Nuclear Governance: Safety*. CIGI Nuclear Energy Futures Special Publication, https://www.cigionline.org

[CR9] Alter KJ, Meunier S (2009). The Politics of International Regime Complexity. Perspectives on Politics.

[CR10] Alter KJ, Raustiala K (2018). The Rise of International Regime Complexity. Annual Social Science.

[CR11] Andonova L (2010). Public-Private Partnerships for the Earth: Politics and Patterns of Hybrid Authority in the Multilateral System. Global Environmental Politics.

[CR12] Andonova L (2017). Governance Entrepreneurs.

[CR13] Ashby WR, von Foerster H, Zopf GW (1962). Principles of the self-organizing system. Principles of Self-Organization.

[CR14] Mukherjee-Reed, A., Reed, D., & Utting, P. (eds) (2012). *Business Regulation and Non-state Actors: Whose Standards?: Whose Development?*. Routledge.

[CR15] Benvenisti E, Downs G (2007). The Empire’s New Clothes: Political Economy and the Fragmentation of International Law. Stanford Law Review.

[CR16] Betsill MM, Bulkeley H (2006). Cities and the Multilevel Governance of Global Climate Change. Global Governance.

[CR17] Betsill M, Dubash N, Paterson M, van Asselt H, Vihma A, Winkler H (2015). Building Productive Links Between the UNFCCC and the Broader Global Climate Governance Landscape. Global Environmental Politics.

[CR18] Biermann, F., & Rakhyun, K. (2020). *Architectures of Global Governance: Institutional Complexity and Structural Transformation* (Cambridge University Press).

[CR19] Biermann, F., Rakhyun, K., Scobie, M., Holloway, J., Mitchell, R., & Abbott, K. W. (2020). Taking Stock and Moving Forward, in *Architectures of Global Governance: Institutional Complexity and Structural Transformation* (Frank Biermann and Rakhyun Kim, eds., Cambridge University Press).

[CR20] Black J (2013). Prudential Regulation of Banks. OECD, International Regulatory Co-Operation: Case Studies.

[CR21] Brown, W. Edith. (1992). International Environmental Issues and the Emergence of a New World Order. *Georgetown Law Journal,**81*(3), 675-710.

[CR22] Bulkeley, Harriet et al. (2014). *Transnational Climate Governance*. Cambridge University Press.

[CR23] Cihon P, Maas MM, Kemp L (2020). Fragmentation and the Future: Investigating Architectures for International AI Governance.

[CR24] Clarke W (2014). Creating the Financial Stability Forum: What Role for Existing Institutions?. Global Society.

[CR25] der Ven V, Hamish SB, Hoffmann M (2017). Valuing the Contributions of Nonstate and Subnational Actors to Climate Governance. Global Environmental Politics.

[CR26] Diefenbach T, Sillince J (2011). Formal and Informal Hierarchy in Different Types of Organization. Organization Studies.

[CR27] Downie C (2020). Steering global energy governance: Who governs and what do they do?.

[CR28] Drezner, D.W. (2013). The tragedy of the global institutional commons, in *Back to basics: State power in the contemporary world*. (Martha Finnemore & Judith Goldstein (eds), Oxford: Oxford University Press, pp. 280–310).

[CR29] Duit A, Galaz V, Eckerberg K, Ebbesson J (2010). Governance, complexity and resilience. Global Environmental Change.

[CR30] Eilstrup-Sangiovanni, M. (2009). Varieties of Cooperation: Government Networks in International Security, in *Networked Politics. Agency, Power and Governance*. (Miles Kahler (ed.), Ithaca, Cornell University Press, pp. 194–227).

[CR31] Eilstrup-Sangiovanni, M., & Westerwinter, O. (2020). Variation and Consequence of Global Governance Complexity. Unpublished Working Paper.

[CR32] Faude, B. (2020a). Breaking Gridlock. How Path Dependent Layering Enhances Resilience in Global Trade Governance. *Global Policy,**11*(4), 448–457.

[CR33] Faude, B. (2020b). International institutions in hard times: how institutional complexity increases resilience. *Complexity, Governance & Networks*, *6*(1), 46–54.

[CR34] Faude B, Fuß J (2020). Coordination or conflict? The causes and consequences of institutional overlap in a disaggregated world order. Global Constitutionalism.

[CR35] Faude, B., & Gehring T. (2017). Regime Complexes as Governance Systems. In: Wayne Sandholtz & Christoher A. Whytock (eds.): *Research Handbook on the Politics of Inernational Law* (Edward Elgar), pp. 176–203.

[CR36] Fioretos O (2011). Historical Institutionalism in International Relations. International Organization.

[CR37] Flonk D, Jachtenfuchs M, Obendiek A (2020). Authority Conflicts in Internet Governance: Liberals versus Sovereigntists?. Global Constitutionalism.

[CR38] Galaz V, Crona B, Osterblom H, Olsson P, Folke C (2011). Polycentric Systems and Interacting Planetary Boundaries – Emerging Governance of Climate Change – Ocean Acidification – Marine Biodiversity. Ecological Economics.

[CR39] Gerhing T, Faude B (2014). A theory of emerging order within institutional complexes: How competition among regulatory international institutions leads to institutional adaptation and division of labor. The International Organizations.

[CR40] Gerhing T, Faude B (2013). The Dynamics of Regime Complexes: Microfoundations and Systemic Effects. Global Governance.

[CR41] Gehring T, Oberthür S (2009). The Causal Mechanisms of Interaction Between International Institutions. European Journal of International Relations.

[CR42] Gómez-Mera L (2017). The Global Governance of Trafficking in Persons: Toward a Transnational Regime Complex. Journal of Human Trafficking.

[CR43] Gordon DJ (2020). Cities on the World Stage: The Politics of Global Urban Climate Governance.

[CR44] Green JF (2013). Order Out of Chaos: Public and Private Rules for Managing Carbon. Global Environmental Politics.

[CR45] Green JF (2014). Rethinking Private Authority.

[CR46] Green JF, Auld G (2017). Unbundling the Regime Complex: The Effects of Private Authority. Transnational Environmental Law.

[CR47] Hale, T. Held D. & Young, K. (2013). *Gridlock: Why Global Cooperation is Failing When We Need It Most.* Polity Press.

[CR48] Hale, Thomas; Held, David (2017): Beyond Gridlock. Cambridge et al.: Polity Press.

[CR49] Hanrieder, T. (2015). WHO Orchestrates? Coping with Competitors in Global Health. In: Kenneth W. Abbott, Philipp Genschel, Duncan Snidal & Bernhard Zangl (eds.): *International Organizations as Orchestrators* (Cambridge), pp. 191–213.

[CR50] Henning, CR., & Tyler P. (2020): Hierarchy and Differentiation in International Regime Complexes: A Theoretical Framework for Comparative Research. Working Paper.

[CR51] Hoffman, Steven J., Clarke BC., & Pearcey, M. (2015). *Mapping Global Health Architecture to Inform the Future*. Chatham House Centre on Global Health Security.

[CR52] Hoffmann, M.J. (2011). *Climate Governance at the Crossroads: Experimenting with a Global Response After Kyoto*. Oxford University Press.

[CR53] Jordan, A., Huitema, D., Schoenefeld, J., van Asselt, H., & Forster, J. (2018). *Governing climate change: Polycentricity in action*, 3–26.

[CR54] Jupille J, Mattli W, Snidal D (2013). Institutional Choice and Global Commerce.

[CR55] Kahler, M. (2020). The Arc of Complex Global Governance: From Organization to Coalition. Unpublished Working Paper.

[CR56] Kahler M (2016). Complex Governance and the New Interdependence Approach. International Political Economy.

[CR57] Karassin, O., & Perez, O. (2020). Public and Private Interactions in Global Environmental Governance. *Elgar Encyclopedia of Environmental Law*, 41–55 (Michael Faure, ed.).

[CR58] Keohane RO, Victor DG (2011). The Regime Complex for Climate Change. Perspectives on Politics.

[CR59] Kreuder-Sonnen C, Zürn M (2020). After fragmentation: Norm collisions, interface conflicts and conflict management. Global Constitutionalism.

[CR60] Lake, D.A. (2020). The Organizational Ecology of Global Governance. *European Journal of International Relations*. Online first.

[CR61] Lake DA (1996). Anarchy, Hierarchy and the Variety of International Relations. International Organization.

[CR62] Lee K (2009). The World Health Organization.

[CR63] Lipscy PY (2015). Explaining institutional change: policy areas, outside options, and the Bretton Woods institutions. American Journal of Political Science.

[CR64] Manulak, M. W., & Snidal, D. (2020). The Supply of Informal Governance: Hierarchy Plus Networks in Global Governance. In *Global Governance in a World of Change,* eds. Michael Barnett, Jon Pevehouse and Kal Raustiala. Cambridge University Press.

[CR65] Marks A, Wouters J (2017). Rule Intermediaries in Global Labor Governance. The Annals.

[CR66] Mattern JB, Zarakol A (2016). Hierarchies in World Politics. International Organization.

[CR67] McConaughey M, Musgrave P, Nexon D (2018). Beyond Anarchy: Logics of Political Organization. Hierarchy and International Structure. International Theory.

[CR68] Morin J-F, Louafi S, Orsini A, Oubenal M (2017). Boundary organizations in regime complexes: a social network profile of IPBES. Journal of International Relations and Development.

[CR69] Morse JC, Keohane RO (2014). Contested Multilateralism. International Organizations.

[CR70] Nye, J.S. Jr. (2014). *The Regime Complex for Managing Global Cyber Activities*. Global Commission on Internet Governance Paper Series #1.

[CR71] Ostrom, E. (2010). Polycentric Systems for Coping with Collective Action and Global Environmental Change. *Global Environmental Change,**20*(4), 550–557.

[CR72] Ostrom V, Tiebout C, Warren R (1961). TheOrganisation of Government in Metropolitan Areas: A Theoretical Inquiry. American Political Science Review.

[CR73] Pattberg P, Widerberg O (2020). Global Sustainability Governance: Fragmented, Orchestrated or Poycentric?. Civitas Europa.

[CR74] Pattberg, P., Chan, S., Sanderink, L., & Widerberg, O. (2018). Linkages: Understanding Their Role in Polycentric Governance. In A. Jordan, D. Huitema, H. van Asselt, & J. Forster (Eds.), *Governing climate change: polycentricity in action?* (pp. 169-187). Cambridge, UK: Cambridge University Press.

[CR75] Pauwelyn, J., Wessel, R., & Wouters, J. (Eds.). (2012).*Informal international lawmaking*. Oxford University Press.

[CR76] Pratt T (2018). Deference and Hierarchy in International Regime Complexes. International Organization.

[CR77] Raustiala K, Victor D (2004). The Regime Complex for Plant Genetic Resources. International Organization.

[CR78] Reinsberg B, Westerwinter O (2021). The global governance of international development: Documenting the rise of multi-stakeholder partnerships and identifying underlying theoretical explanations. International Organizations.

[CR79] Rixen, T., & Viola, L. A. (2020). Indirect Governance in Global Financial Regulation, in Kenneth W. Abbott et al., eds., *The Governor’s Dilemma. Indirect Governance Beyond Principals and Agents*. Oxford.

[CR80] Roger, Charles (2020): The Origins of Informality. Why the Legal Foundations of Global Governance are Shifting, and Why It Matters. Oxford, UK: Oxford University Press.

[CR81] Ruggie JG (2014). Global Governance and “New Governance Theory”: Lessons from Business and Human Rights. Global Governance.

[CR82] Shanks C, Jacobson HK, Kaplan JH (1996). Inertia and change in the constellation of international governmental organizations, 1981–1992. International Organization.

[CR83] Slaughter A-M (2004). A New World Order.

[CR84] Stein AA (1982). Coordination and collaboration: regimes in an anarchic world. International Organization.

[CR85] Stone, R. (2011). *Controlling Institutions. International Organizations and the Global Economy*. Cambridge: Cambridge University Press.

[CR86] Streeck, W., & Thelen, K. (2005). Introduction. Advanced Political Economies. Pp. 3–39 in Wolfgang Streeck and Kathleen Thelen (eds.): Beyond Continuity: Institutional Change in Advanced Political Economies. Oxford University Press.

[CR87] Tallberg, J., Sommerer, T., Squatrito, T., & Jönsson, C. (2013). *The opening up of international organizations*. Cambridge University Press.

[CR88] Vabulas F, Snidal D (2020). Informal IGOs as Mediators of Power Shifts. Global Policy.

[CR89] Vabulas F, Snidal D (2013). Organization without Delegation: Informal Intergovernmental Organizations (IIGOs) and the Spectrum of Intergovernmental Arrangements. International Organizations.

[CR90] Viola, L. A. (2015). The Governance Shift: From Multilateral IGOs to Orchestrated Networks. In: Mayntz, Renate (ed.): Negotiated Reform: TheMultilevel Governance of Financial Regulation. Frankfurt-on-Main: Campus. pp. 17–36.

[CR91] Westerwinter, O. (Forthcoming). *Contextual Design: Existing International Institutions and New Transnational Governance*.

[CR92] Westerwinter, O. (2020). Explaining IGO Participation in Transnational Governance Initiatives. Unpublished Working Paper.

[CR93] Westerwinter O, Abbott KW, Biersteker T (2021). Informal governance in world politics. International Organizations.

[CR94] Widerberg, O., Patterg, P., & Kristensen, K. (2016). Mapping the institutional architecture on global climate change governance. Version 2.0.Report number: R-16/02. Institute for Environmental Studies (IVM) at VU University Amsterdam.

[CR95] Young O (2002). The Institutional Dimensions of Environmental Change: Fit.

[CR96] Young O (1996). Institutional Linkages in International Society. Global Governance.

[CR97] Zeitlin J, Overdevest C (2020). Experimentalist interactions: Joining up the transnational timber legality regime.

